# Type 1 Brugada Pattern May Be Provoked by Ajmaline in Some Healthy Subjects: Results From a Clinical Trial

**DOI:** 10.1161/CIRCULATIONAHA.123.067223

**Published:** 2024-05-20

**Authors:** Bode Ensam, Chiara Scrocco, David Johnson, Yanushi D. Wijeyeratne, Rachel Bastiaenen, Belinda Gray, Chris Miles, Yael Ben-Haim, Stathis Papatheodorou, Sanjay Sharma, Michael Papadakis, Brian Devine, Peter W. Macfarlane, Elijah R. Behr

**Affiliations:** Cardiology Section and Cardiovascular Clinical Academic Group, St. George’s University Hospitals NHS Foundation Trust, and Institute of Molecular and Clinical Sciences, St. George’s University of London, UK (B.E., C.S., D.J., Y.D.W., R.B., B.G., C.M., Y.B.-H., S.P., S.S., M.P., E.R.B.).; Electrocardiology Group, School of Health and Wellbeing, University of Glasgow, Scotland (B.D., P.W.M.).; Faculty of Medicine and Health, University of Sydney, NSW, Australia (B.G.).

**Keywords:** death, sudden

The diagnostic utility of a drug-induced type 1 Brugada pattern (DI-T1BP) has been much debated, and in isolation, the DI-T1BP is no longer considered indicative of Brugada syndrome (BrS).^1,2^ Furthermore, common and rare genetic variation has been associated with BrS and the electrocardiographic response to sodium channel blocker provocation (SCBP),^3,4^ suggesting a complex susceptibility for the DI-T1BP.

We sought to understand the clinical utility of SCBP and the electrocardiographic response to ajmaline in systematically recruited healthy White subjects. We then explored the relationship with relevant common and rare genetic variation. The study was approved by the Southeast London Research Ethics Committee (16/LO/2173), and all subjects provided written consent to participate.

After completing a comprehensive medical questionnaire and baseline cardiovascular assessment, 100 asymptomatic White subjects (mean age, 26.83±8.01 years; 52% male) without a family history of sudden death (maximum pre-SCBP Shanghai score of 0^2^) and with normal resting ECG and transthoracic echocardiogram underwent SCBP (Figure). All subjects received 100% of target dose (1 mg/kg up to a maximum of 100 mg) over 5 minutes. A continuous ECG was acquired during SCBP in the standard and high right precordial lead configuration. Quantitative electrocardiographic variables were analyzed with automated electrocardiographic software and based on an average beat for each lead. Peak drug effect, denoted by maximal QRS duration, was observed at 6 minutes 00±80 seconds. In 3 subjects, the DI-T1BP was observed (Figure). Subsequent high right precordial lead 12-lead Holter monitoring did not reveal a spontaneous T1BP.

**Figure.**
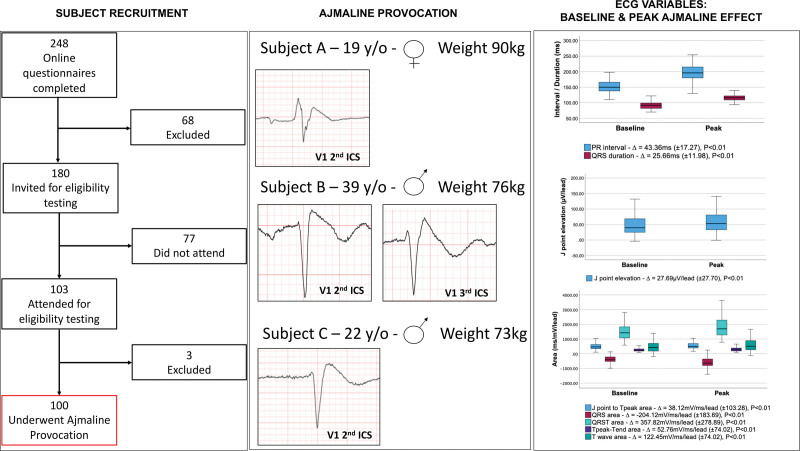
**Flow diagram indicates that of 168 respondents, 68 were excluded on medical or family history and 3 on baseline ECG.** ECGs show the drug-induced type 1 Brugada pattern in 3 subjects. Box-and-whisker plot of significant baseline and peak ajmaline effects on electrocardiographic variables. ICS indicates intercostal space; and y/o, years old.

Qualitative and quantitative electrocardiographic variables were recorded at baseline and at peak drug effect, and the latter was expressed as peak minus baseline (∆) values. The Kolmogorov-Smirnov test was used to test the distribution of data. The Mann-Whitney test was used to compare nonnormally distributed continuous variables, which are reported as median (quartile 1–3). Normally distributed continuous variables were analyzed with an independent-sample *T* test and are reported as mean±SD. A paired sample *t* test was used to analyze quantitative electrocardiographic variables at baseline and peak drug effect in the overall cohort, and an independent-sample *T* test was used to analyze electrocardiographic variables between those with the DI-T1BP (positive) and those without it (negative). A value of *P*<0.05 was considered significant in all cases.

In the overall cohort, significant increases in ∆QRS duration (∆25.66±11.98 milliseconds; *P*<0.01) and ∆PR interval (∆ 43.36±17.27 milliseconds; *P*<0.01) were observed. There was also a significant increase in mean J-point elevation in the right precordial leads (standard and high right precordial lead; ∆27.69±27.70 µV/lead; *P*<0.01). Net area, defined as the difference between positive and negative QRST area with respect to the isoelectric line, was measured in the standard and high right precordial leads and was either net positive or net negative, depending on the net direction with reference to the isoelectric line. A net negative increase in QRS area (∆−204.12±183.69 mV/ms per lead; *P*<0.01) was observed, whereas J-point to Tpeak area (∆38.12±103.28 mV/ms per lead; *P*<0.01), T-wave area (∆122.45±225.60 mV/ms per lead; *P*<0.01), and Tpeak to Tend area (∆52.76±74.02 mV/ms per lead; *P*<0.01) all demonstrated a net positive medium increase in area. There were no significant differences in the baseline, peak drug effect, or ∆electrocardiographic variables between DI-T1BP–positive and –negative subjects.

Eighty-eight of 100 subjects with suitable DNA, including the 3 cases with DI-T1BP, were genotyped with an Illumina GSA 3.0 array and underwent *SCN5A* sequencing. We did not identify any pathogenic or likely pathogenic variants or variants of uncertain significance in *SCN5A*, nor did we find any association with the DI-T1BP and the previously reported BrS polygenic risk score.^4,5^

In summary, we observed the DI-T1BP in 3 of 100 (3%) systematically recruited healthy White subjects with normal clinical testing and without a family history of sudden death (Figure). We observed significant effects of ajmaline on PR interval and QRS duration, the area beyond the J point and ST amplitude (Figure). The effect on QRS duration was likely to be responsible for much of the effect on QRS area. These observations suggest that the effects of ajmaline may be useful in providing a quantitative element to the diagnosis of BrS in patients undergoing SCBP. We did not identify any correlation with rare *SCN5A* variations or with the previously reported BrS polygenic risk score. This is likely due to the small cohort size.

Our data suggest that an isolated DI-T1BP in the absence of clinical or genetic features supportive of BrS or a family history of sudden death may not be diagnostic of the condition. A model incorporating clinical, quantitative electrocardiographic and genomic data will be required for an evidence-based diagnosis of concealed BrS.

## ARTICLE INFORMATION

### Acknowledgments

The authors acknowledge Dr Velislav Batchvarov, MD (deceased), Cardiology Clinical Academic Group, St. George’s University Hospitals NHS Foundation Trust, and Institute of Molecular and Clinical Sciences, St. George’s University of London, London, UK.

### Sources of Funding

Dr Ensam is supported by the Robert Lancaster Memorial Fund sponsored by McColl’s, Cardiac Risk in the Young UK, and the British Heart Foundation (PG/19/58/34581). B. Gray is the recipient of a NHMRC Early Career Fellowship (No. 1122330). Dr Scrocco is supported by the Robert Lancaster Memorial Fund sponsored by McColl’s and the British Heart Foundation (PG/15/107/31908).

### Disclosures

None.
